# Evaluating racial disparities in cancer patient-provider communication about cannabis in a state without a legal cannabis marketplace

**DOI:** 10.1007/s00520-024-09131-9

**Published:** 2025-01-08

**Authors:** Amelia V. Wedel, Kyle J. Walters, Rachel L. Tomko, Alana M. Rojewski, Erin A. McClure

**Affiliations:** 1 Department of Counseling Psychology, Santa Clara University, 455 El Camino Real, Santa Clara, CA 95050 USA; 2 Department of Individual, Family, and Community Education, University of New Mexico, Albuquerque, NM USA; 3 Department of Psychiatry and Behavioral Sciences, Medical University of South Carolina, Charleston, SC USA; 4 Hollings Cancer Center, Medical University of South Carolina, Charleston, SC USA; 5 Department of Public Health Sciences, Medical University of South Carolina, Charleston, SC USA

**Keywords:** Cannabis, Race, Black patients, Patient-physician communication

## Abstract

**Purpose:**

Cancer survivors in a state with no legal access to cannabis may be hesitant to discuss their cannabis use with providers, particularly in light of legal consequences which disproportionately affect certain racial groups. This study examined potential racial disparities in the relationship of cannabis use status with patient-provider discussions of and attitudes toward cannabis in a state where there is no legal cannabis marketplace.

**Methods:**

Survivors of cancer (*N* = 1003, *M*_age_ = 62.36; 13% Black/African-American; 41% male) completed a cross-sectional survey. Weight-adjusted regressions examined racial differences in the relationship between cannabis use status with (a) comfort and discussion of cannabis with providers, and (b) beliefs about impact of legalization on patients’ and providers’ comfort discussing cannabis.

**Results:**

No racial differences were observed in rates of cannabis use or discussion, and patients who used cannabis were more comfortable discussing cannabis. Black patients who had used cannabis reported the greatest comfort discussing cannabis with providers, but also the greatest perceived improvement in comfort in the event of legalization.

**Conclusions:**

Results highlight comfort and willingness to discuss cannabis with cancer care providers, particularly among Black patients who already use cannabis, which was not the hypothesized direction of findings. Further work is needed to inform recommendations for provider-led communication about cannabis.

Cannabis use is common among cancer survivors, with prevalence estimates ranging from 8 to 40% [6, 16], even in states where there is no legal market (i.e., South Carolina; [18]. Patients with cancer may use cannabis in part to manage cancer- or treatment-related symptoms, such as sleep difficulties, pain, nausea, anxiety or depression [8, 26]. Altogether, cannabis is viewed as helpful and is sought-out among patients with cancer for its potential benefits [20], even after accounting for its potential health harms (i.e., drowsiness, dizziness, or deleterious effects on mental health,[7, 23], necessitating open communication between patients and providers about cannabis use.

While cannabis use is common among patients with cancer, data are limited regarding patient comfort with discussing non-recommended and non-authorized cannabis use [13]. Qualitative research shows that patients with legal access to cannabis are comfortable discussing cannabis use with providers and are often curious to learn more [5, 20]. By contrast, patients with cancer who use cannabis obtained outside of the legal market may be hesitant to disclose or discuss their cannabis use with their healthcare team for fear of legal consequences for themselves or their provider, or due to concerns that their cannabis use might be either stigmatized or unsupported [25, 28]. Despite this discrepancy, quantitative evidence is lacking in this area, and it is unclear how willing patients with limited or restricted legal access may be to disclose and discuss cannabis use with their providers.

Patients’ comfort with disclosing illicit cannabis use to their providers may be further complicated by racial disparities in the patient-provider relationship. Race is a nonbiological, socially constructed categorization of people based on perceived shared physical traits [2], the social nature of which serves to maintain existing sociopolitical hierarchy. In healthcare settings, this can be expressed as discrimination and racism that, even when enacted subconsciously, have been associated with differences in life expectancy, poorer health outcomes, and poorer overall quality of care [4]. Socioecological theory posits that the patient-provider relationship is the result of a complex interplay between patients’ experiences and expectancies of discrimination, providers’ implicit and explicit biases, and systemic factors which can impact patients’ health literacy and access to care [19]. The impact of these interrelated factors on the patient-provider relationship may be particularly salient among non-Hispanic Black patients: compared with non-Hispanic White and Hispanic groups, Black patients with cancer tend to report lower quality relationships with their providers, especially in domains related to respect and patient-provider communication [27]. This trend toward poor patient-provider communication among Black patients may be especially relevant for issues pertaining to cannabis use given disproportionately high rates of legal repercussions for cannabis use among Black individuals in states without legal cannabis markets [1]. Across studies, Black patients with cancer demonstrate the highest prevalence of medical cannabis use across racial groups [12, 21], yet report the greatest barriers to using cannabis as part of their cancer treatment [22]. Altogether, evidence suggests that Black patients may be particularly reticent to discuss or disclose cannabis use with their healthcare providers, potentially exacerbating any existing differences in willingness to discuss cannabis as an option based on past (or current) cannabis use.

To address important gaps in the literature pertaining to racial disparities in the patient-provider relationship and discussion of cannabis use, we conducted an exploratory analysis from a cross-sectional survey conducted at the National Cancer Institute (NCI)-designated Hollings Cancer Center (HCC) at the Medical University of South Carolina (MUSC) Health system in South Carolina (SC). Results from this survey, including prevalence estimates of cannabis use across the patient population, have been reported elsewhere [18], which demonstrated 26% of cancer patients at HCC used cannabis since their diagnosis. Notably, in SC, access to cannabis, specifically products with greater than 0.3% ∆^9^-tetrahydrocannabinol (THC), is illegal (with few exceptions, including low-THC and high cannabidiol [CBD] preparations and prescription of CBD for epilepsy; [11]). This analysis aimed to examine the relationship of race and cannabis use status with the following outcomes: (1) patients’ comfort with discussing cannabis use with their provider, (2) providers’ recommendations and discussion of cannabis use with the patient, (3) potential changes to patients’ and providers’ comfort discussing cannabis use resulting from legalization. Across outcomes, it was hypothesized that (1) race (as a proxy for systemic factors, namely racism) would adversely affect patient-provider communication, and (2) racial disparities would be exacerbated by cannabis use since cancer diagnosis, such that Black patients who had used cannabis since diagnosis would be least comfortable discussing cannabis with providers.

## Methods

### Survey administration and sampling

This cross-sectional survey was administered to patients at the MUSC Hollings Cancer Center in Charleston, SC, USA from June 2021 through April 2022. This survey was part of the NCI Cannabis Supplement [18], which was awarded to 12 P30 cancer centers across the US, including HCC (P30CA138313, PI Dubois, Supplement PI, McClure). Probability sampling methods were used to sample eligible HCC cancer patients. To be eligible, participants had to meet the following criteria: (1) age 18 or older, (2) able to read and understand English, and (3) must have received a cancer diagnosis and/or cancer care from 2018 through 2020 at the HCC or an MUSC clinic (in Charleston, SC). Patients were excluded from the randomly selected sample who were known to be deceased or if they had opted out of research contact. Of the 8000 patients initially sampled, 13.4% responded, resulting in a total sample of 1048 participants who consented and completed the 10–30 min survey and were compensated with a $20 Amazon gift card; of these, 1003 participants were included in the current analyses (after excluding *n* = 15 participants with missing data on key variables and *n* = 30 participants belonging to racial groups other than Black or White).

### Ethical considerations

All procedures were approved by the MUSC IRB in accordance with the Declaration of Helsinki; in brief, all participants provided informed consent, were able to withdraw from participation at any time, and had their information protected via anonymization of all responses. A comprehensive description of procedural details and results have been previously reported [18].

### Measures

#### Demographics

Demographics were extracted from the MUSC electronic medical record (EMR), including age, sex assigned at birth (0 = *Female*, 1 = *Male*), ethnicity (0 = *Non-Hispanic*, 1 = *Hispanic*), and race. Race was provided from the EMR with categories including: White, Black/African American, Asian, and Other or Unknown. Given small numbers of HCC patients identifying as a race other than White or Black/African American, and given the focus of this analysis on racial disparities among Black vs. White patients, we excluded from analyses participants who reported other racial categories. We acknowledge this as a limitation to our dataset and analyses. In order to ensure a sample representative of the patient population and to account for expected under-enrollment of certain subgroups, these four demographic variables from the EMR were used to weight the sample back to the patient population so that survey responses more closely approximated the SC cancer patient population receiving care at the MUSC HCC.

#### Cannabis use

Cannabis was defined in the survey instrument as “any of the following: marijuana, cannabis concentrates, edibles, lotions, ointments, tinctures containing cannabis, CBD-only products, pharmaceutical or prescription cannabinoids (e.g., Dronabinol, Nabilone, Marinol, Syndrose, Cesamet), other products made with cannabis.” All participants reported their use of cannabis prior to and since diagnosis (use prior to diagnosis, since diagnosis, use during cancer treatment, use after treatment, current use). For analyses, cannabis use was dichotomized (0 = *No use since diagnosis*, 1 = *Any cannabis use since diagnosis*).

#### Patient-provider discussions of cannabis use

All participants were administered several questions pertaining to their comfort discussing cannabis use with their doctor and medical team. First, participants responded on a 4-point Likert scale (1 = *Extremely uncomfortable* to 4 = *Extremely comfortable*) to the question “How comfortable would you feel talking with your healthcare providers about cannabis?” Participants were also asked whether they had discussed cannabis for cancer-related symptoms with their provider (1 = *Yes*; 0 = *No*).

Those who reported cannabis use since diagnosis were also asked whether their doctor or another healthcare provider knew of their cannabis use at any point during their treatment (1 = *Yes*; 0 = *No*).

#### Legalization-related changes to provider discussion of cannabis use

All participants answered two questions along a 5-point Likert scale (1 = *Strongly disagree* to 5 = *Strongly agree*) regarding increased comfort having conversations with their providers if cannabis were legal. Specifically, participants responded to an item asking about their *own* comfort talking to a provider about cannabis if it were legal, and to an item asking about their perception of their *doctor*’*s* comfort talking about cannabis to them if it were legal.

### Statistical methods

#### Weighting procedures

A total of 1048 participants started the survey; weighting procedures were based on 1036 respondents, as 12 participants were excluded due to high rates of missing data. Three participants did not provide information about their cannabis use and were thus excluded from results (*n* = 1033). Due to low representativeness of racial groups other than White (*n* = 844) and Black/African-American (*n* = 112) in the unweighted study sample, respondents who were Asian (*n* = 5) and whose race was other or unknown (*n* = 25) were excluded from the sample, resulting in a final sample of 1003 participants. All results are presented with the included sample for this analysis (*N* = 1003). Sample weights were constructed based on selection probabilities, non-response adjustment, and post-stratification via ranking to match the sample to known subgroup proportions in the population (see [18]),extreme sample weights (> 2 SDs above mean weight) were winsorized to the 95th percentile. The final sample proportions are demographically similar to the proportions presented in the population totals from the HCC’s sampling frame, with two exceptions: the current sample has a lower proportion of patients 75 years and older (14% in our sample, versus 22% in the HCC sampling frame), and the current sample has a greater proportion of White respondents (87% in our sample, versus 75% in the HCC sampling frame) relative to Black respondents (13% in our sample, versus 20% in the HCC sampling frame). Both before and after weighting procedures, Black participants were more likely to be female (χ^2^(1) = 107.13, *p* < 0.001) and younger (*t*(973) =  − 20.52, *p* < 0.001) than White participants.

#### Analyses

Weighted descriptive percentages are presented. Weight-adjusted Chi-square tests were conducted to examine differences in demographics, cannabis use status, and discussion of cannabis use with providers. Weighted logistic and linear regression analyses were conducted using the *survey* and *svyVGAM* packages in R version 4.2.2 [14, 15, 24], which specify design characteristics using sampling units and weights. Specifically, weighted logistic regression was used to examine (a) provider recommendations to use cannabis, (b) actual patient-provider discussion of cannabis use, and (c) provider knowledge of patient cannabis use (for patients who reported cannabis use since diagnosis, who were the only participants to whom this item was administered; *n* = 274). Weighted linear regressions were used to examine (a) patient comfort discussing cannabis with healthcare providers, (b) perceived changes to patient comfort with cannabis discussions in the event of legalization, and (c) perceived changes to provider comfort with cannabis discussions in the event of legalization. For all analyses, a product interaction term (cannabis use status x race) was included, and marginal effects of cannabis use status (i.e., endorsing cannabis use since diagnosis) on each racial group were subsequently calculated to interpret the effects of the interaction term [17]. The finite population correction factor was used for all standard error estimates due to the size of the sample relative to the target population (> 5%). All analyses controlled for sex and age.

Across all analyses, Black participants were hypothesized to be less willing and/or comfortable to discuss cannabis with their healthcare provider and to perceive their provider(s) as less willing to discuss cannabis with them; moreover, cannabis use status was hypothesized to moderate this relationship, such that this relationship would be strengthened among those who had used cannabis since their diagnosis.

## Results

### Differences by cannabis use status

Sample descriptive statistics are reported in Table 1 (*N* = 1003). No significant differences in cannabis use since cancer diagnosis were observed across any sociodemographic category. Altogether, 12% of participants reported that they had discussed using cannabis for cancer symptoms with their healthcare provider, though this percentage was higher (30%) among participants who reported using cannabis since diagnosis. Compared to participants who had not used any cannabis since diagnosis, those who had used cannabis since diagnosis reported greater perceived comfort discussing cannabis use were it legalized, both on the part of themselves (*t*(551) = 5.04, *p* < 0.001) and on the part of their providers (*t*(578) = 5.85, *p* < 0.001).

**Table 1:** Descriptive statistics for full sample

Variable (range)	Whole sample	No cannabis use since diagnosis	Cannabis use since diagnosis	Statistical test
	*N* = 1003	*n* = 716	*n* = 287	
	*M* (SD) or %	*M* (SD) or %	*M* (SD) or %	
Sociodemographics				
Age (20–92)	62.36 (13.15)	64.22 (12.71)	57.69 (13.12)	*t*(493) = 7.08***
Male sex	41%	41%	41%	χ^2^(1) = 0.00
White race	87%	87%	89%	χ^2^(1) = 1.16
Black or African American race	13%	13%	11%	χ^2^(1) = 1.16
Hispanic ethnicity	1%	2%	1%	χ^2^(1) = 0.00
Provider discussion items				
Comfort talking to healthcare providers about cannabis (1–4)	3.13 (1.02)	3.08 (1.02)	3.21 (1.01)	*t*(536) = − 1.77
Discussion of cannabis for cancer symptoms (% Yes)	12%	4%	30%	χ^2^(1) = 138.17***
Provider recommendation of cannabis use for cancer symptoms (% Yes)	4%	1%	10%	χ^2^(1) = 44.19***
Healthcare providers’ knowledge of cannabis use during treatment (% Yes)	–	–	41%	–
Perceived patient comfort if cannabis were legalized (1–5)	3.65 (1.27)	3.56 (1.28)	3.99 (1.18)	*t*(551) = − 5.04***
Perceived provider comfort if cannabis were legalized (patient-reported; 1–5)	3.83 (1.04)	3.76 (1.07)	4.16 (0.93)	*t*(578) = − 5.85***

*N* = 1003

***p < 0.001

### Comparing racial differences in patient-provider discussion of cannabis use

**Fig. 1:**
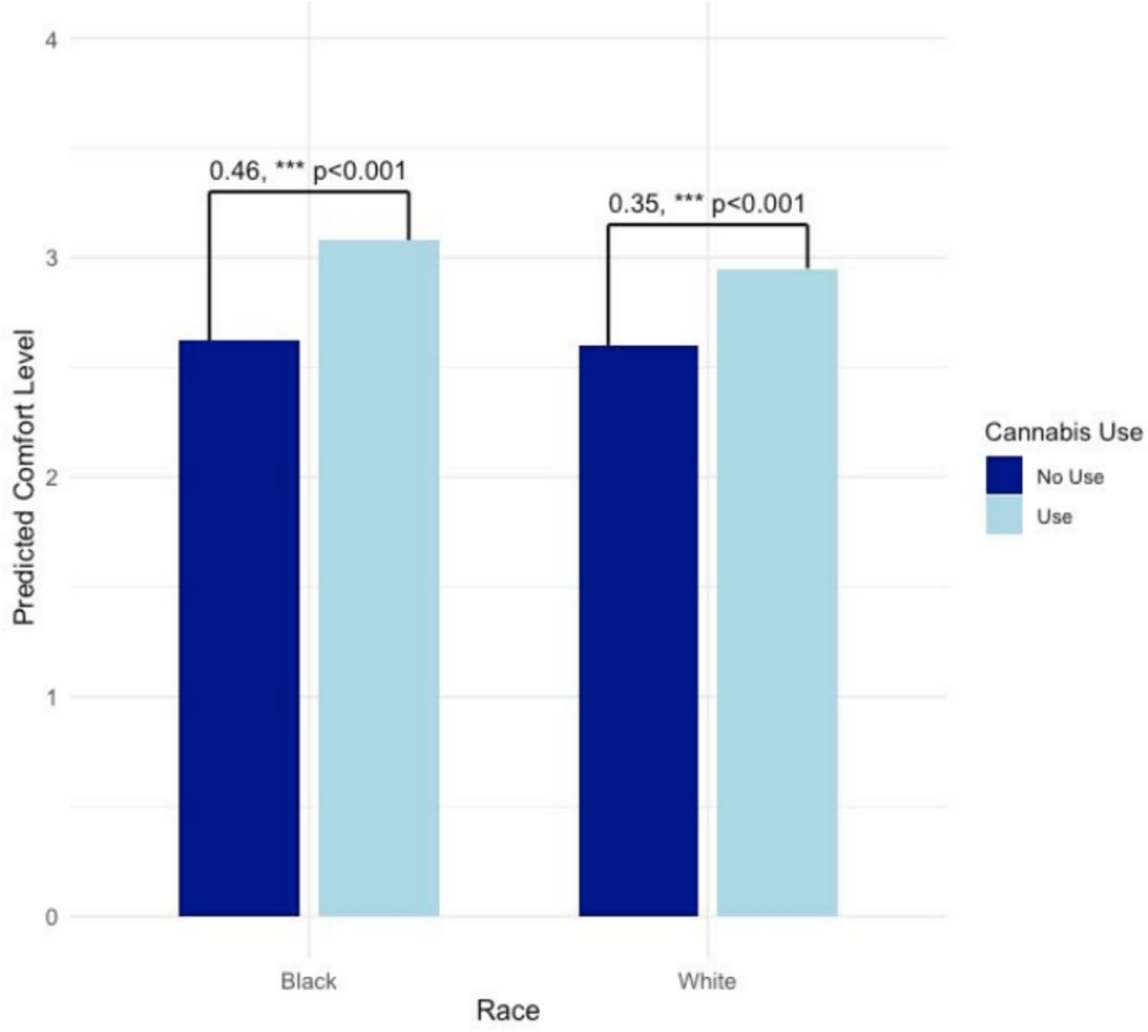
Cancer patients’ comfort discussing cannabis by race and use. *N* = 1003. Bars represent marginal effects for each racial group

Within the sample of White and Black patients (*N* = 1003), results of a weighted linear regression showed that using cannabis since diagnosis significantly moderated the relationship of race with comfort discussing cannabis use with healthcare providers (*B* = 0.35, *p* < 0.001), such that there was a steeper increase in comfort among those using cannabis for Black/African-American participants who reported cannabis use since diagnosis than for White participants who reported cannabis use (Table 2, column 1; Fig. 1).

**Table 2:** Patient comfort, discussion, and provider recommendations and knowledge of patient cannabis use

	How comfortable would you feel talking with your healthcare providers about cannabis?	At any time since your cancer diagnosis, has your doctor or another healthcare provider recommended that you use cannabis?	Have you discussed using cannabis for your cancer symptoms with a healthcare provider?	At any time since your cancer diagnosis, did your cancer doctor or team know you were using cannabis?
	*N* = 1003	*N* = 1003	*N* = 1003	*n* = 274
	*B* (SE)	OR (95% CI)	OR (95% CI)	OR (95% CI)
Intercept	2.60 (0.04)***	0.06 (0.04, 0.09)***	0.15 (0.12, 0.19)***	2.06 (0.62, 6.80)
Cannabis use since diagnosis (ref = none)	0.11 (0.02)***	8.63 (7.04, 10.58)***	9.79 (8.71, 10.99)***	–
Black race (ref = White race)	0.02 (0.03)	1.03 (0.63, 1.66)	1.22 (0.95, 1.56)	3.83 (1.64, 8.97)**
Sex (ref = female)	0.01 (0.02)	0.97 (0.80, 1.22)	1.35 (1.20, 1.52)***	1.27 (0.73, 2.20)
Age	0.01 (0.00)***	0.97 (0.97, 0.98)***	0.98 (0.97, 0.98)***	0.98 (0.96, 0.99)*
Marginal effects				
Cannabis use since diagnosis (ref = none)				
Black race	0.46 (0.05)***	2.39 (1.23, 4.69)*	10.73 (10.44, 11.01)***	-
White race	0.35 (0.05)***	0.28 (0.14, 0.55)***	1.12 (0.82, 1.52)	-

*N* = 1003. Main effects are presented first, and marginal effects of cannabis use status for different racial groups are presented second. For all analyses, including marginal effects analyses, the referent group for cannabis use since diagnosis is “no cannabis use since diagnosis.” The item “At any time since your cancer diagnosis, did your cancer doctor or team know you were using cannabis?” was administered only to participants who reported using cannabis since their diagnosis, preventing analysis of main effects of cannabis use status or moderation by cannabis use status, and resulting in a lower sample size (*n* = 274)

**p < 0.01, ***p < 0.001

Likewise, results of a weighted logistic regression showed that cannabis use since diagnosis significantly moderated the relationship of race with whether a healthcare provider had recommended cannabis use to the patient. Specifically, providers were more likely to have recommended cannabis to White patients who had used cannabis since diagnosis than to Black participants who had used cannabis since diagnosis (Table 2, column 2).

However, cannabis use since diagnosis did not moderate associations of race with odds of actual discussion of cannabis use with a healthcare provider (*p* = 0.640; Table 2, column 3). After removing the interaction term to test main effects, cannabis use since diagnosis (OR = 10.01 [95% CI = 8.94, 11.21], *p* < 0.001) and Black race (OR = 1.29 [95% CI = 1.12, 1.50], *p* < 0.001) were associated with significantly higher odds of discussing cannabis with their provider.

Among participants who reported using cannabis since diagnosis (*n* = 274), inconsistent with hypotheses, Black participants reported significantly *higher* odds of their cancer doctor or team knowing they were using cannabis (Table 2, column 4).

### Legalization-related changes to comfort with provider discussion of cannabis use

**Fig. 2:**
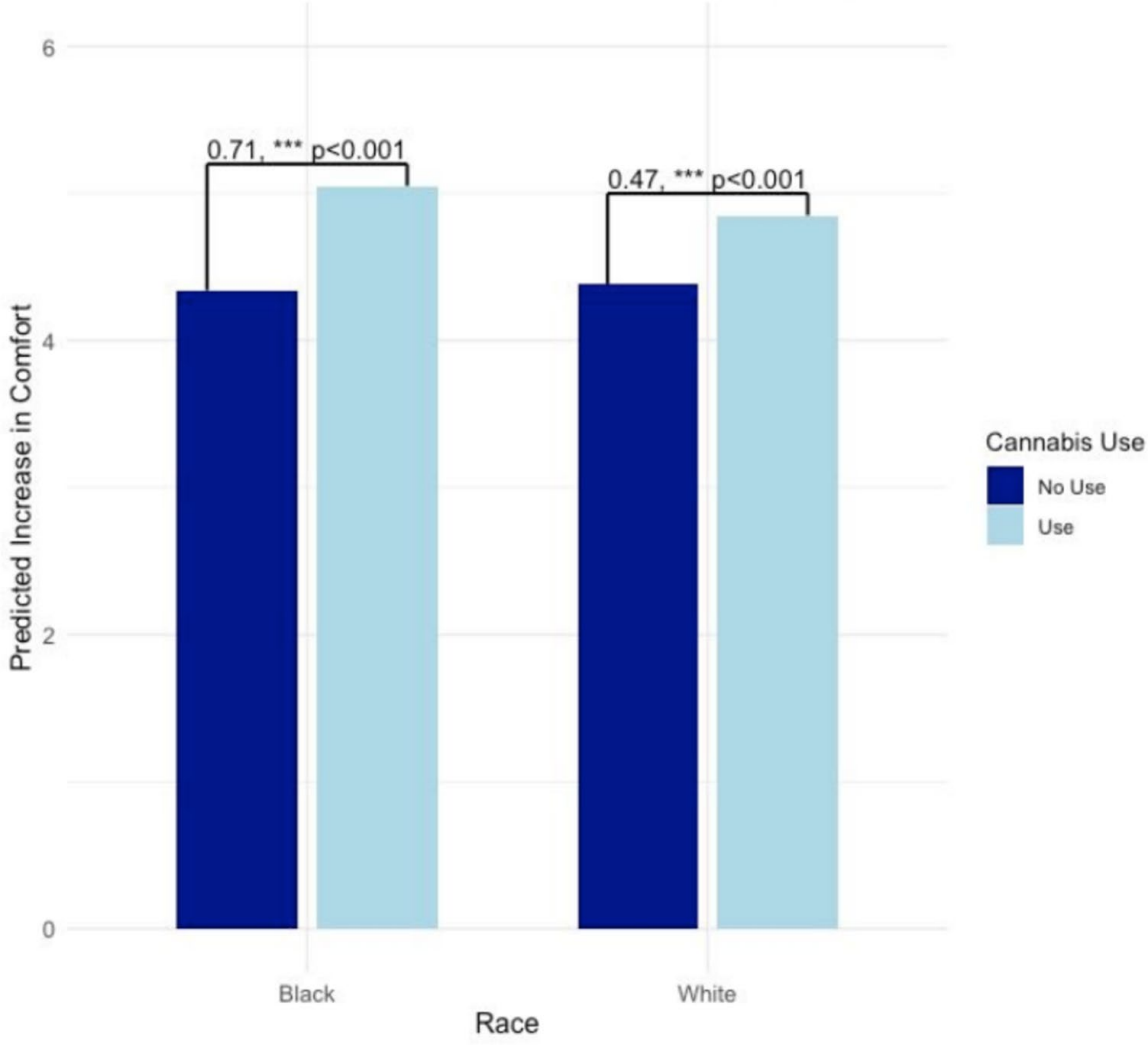
Cancer patients’ perceived changes to their own comfort discussing cannabis in case of legalization. *N* = 1003. Bars represent marginal effects for each racial group

Hypothetical changes in cannabis perceptions and behavior based on legalization in South Carolina were examined among all participants, regardless of cannabis use status. Models examined changes in comfort discussing cannabis with providers following legalization (*B* = 0.47, *p* < 0.001; Table 3, column 1 and Fig. 2). Regarding patient perceptions of their *own* comfort discussing cannabis with their doctor, cannabis use since diagnosis was found to moderate the relationship of race with comfort, such that Black participants evidenced a stronger relationship between their cannabis use and perceived improvements to discussing cannabis if it were legal.

**Table 3:** Legalization-related changes to comfort with provider discussion of cannabis use

	I would feel more comfortable talking to my doctor about cannabis if it were legal in South Carolina	My doctor would feel more comfortable talking about cannabis to me if it were legal in South Carolina
	B (SE)	B (SE)
Intercept	4.38 (0.05)***	4.57 (0.04)***
Cannabis use since diagnosis (ref = none)	0.24 (0.02)***	0.23 (0.02)***
Black race (ref = White race)	− 0.04 (0.04)	− 0.37 (0.03)
Sex (ref = female)	− 0.10 (0.02)***	− 0.12 (0.02)***
Age	− 0.01 (0.00)***	− 0.01 (0.00)***
Marginal effects		
Cannabis use since diagnosis (ref = none)		
Black race	0.71 (0.05)***	0.69 (0.05)***
White race	0.47 (0.05)***	0.46 (0.05)***

*N* = 1003. Main effects are presented first, and marginal effects of cannabis use status for different racial groups are presented second. For all analyses, including marginal effects analyses, the referent group for cannabis use since diagnosis is “no cannabis use since diagnosis”

***p < 0.001

**Fig. 3:**
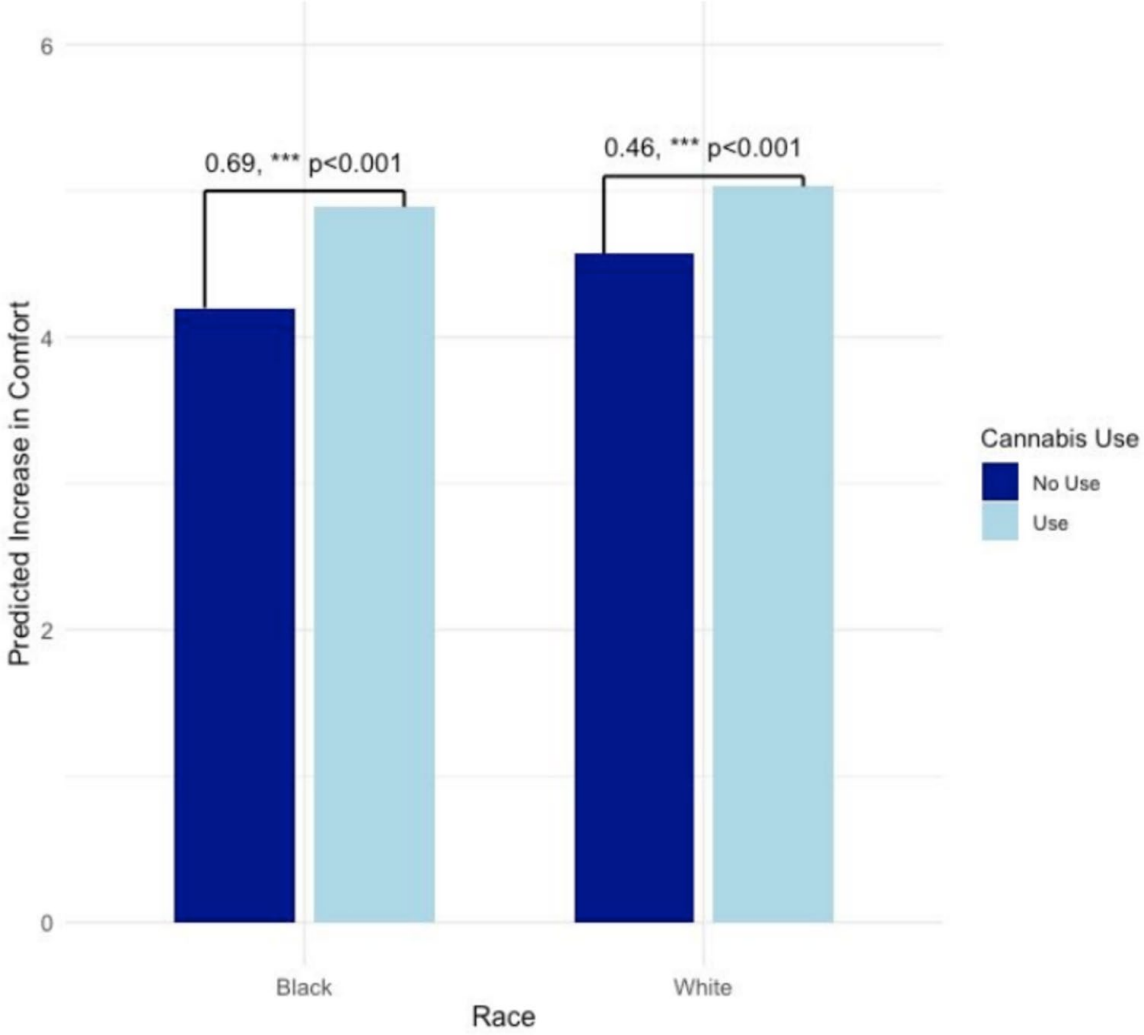
Cancer patients’ perceived changes to providers’ comfort discussing cannabis in case of legalization. *N* = 1003. Bars represent marginal effects for each racial group

Regarding patient perceptions of *providers*’ comfort discussing cannabis if it were legal, cannabis use since diagnosis was likewise found to moderate associations of race with doctors’ comfort discussing cannabis (*B* = 0.46, *p* < 0.001; Table 3, column 2 and Fig. 3). As with their own comfort, Black participants evidenced a stronger relationship between their cannabis use and perceived improvements to their providers’ comfort discussing cannabis if it were legal.

## Discussion

This cross-sectional survey conducted among an oncology population in a state without a legal cannabis market examined the role of cannabis use status and race (Black compared to White participants) in patients’ attitudes toward and openness to discussing cannabis with healthcare providers. Contrary to hypotheses that Black participants, and particularly Black participants who used cannabis, would be hesitant to discuss cannabis with healthcare providers, findings show greater comfort discussing cannabis among Black cancer patients who used cannabis. Findings did not demonstrate any racial differences in cannabis use rates or in endorsement of discussion of cannabis use with providers. Further, Black cancer patients who use cannabis feel more comfortable discussing cannabis, would feel even more comfortable discussing cannabis with providers if cannabis were legalized in SC, and anticipate that their providers would likewise feel more comfortable discussing cannabis if it were legalized in SC.

Regardless of race, findings showed generally greater comfort discussing cannabis with providers among patients using cannabis since their cancer diagnosis, as well as greater likelihood of discussing cannabis with providers. Greater comfort discussing cannabis among patients using cannabis (as opposed to those not using cannabis) is not surprising given that those who used cannabis since diagnosis may be more likely to disclose this behavior to their providers, thus initiating the conversation. However, previous results from our survey found that 17% of the sample not using cannabis considered using since diagnosis, but did not due to a number of reasons including legal consequences [18]. This suggests there is a proportion of patients who are open to cannabis use, but may experience some discomfort in discussing this with providers. Additionally, and contrary to hypotheses, Black participants who used cannabis evidenced *greater* comfort discussing cannabis with providers, though there was no apparent effect of race on actual discussion of cannabis. This unexpected finding may be explained in part by previous results that Black patients tend to see honesty and self-disclosure as a form of necessary self-advocacy when meeting with providers [3]. Alternatively, this may suggest strong patient-provider relationships and communication among our patient population and center providers.

Participants in this study generally perceived that both their comfort and the comfort of their providers’ discussion of cannabis would improve if cannabis were to be legalized in SC. With respect to increases in their own comfort, Black patients who had used cannabis since diagnosis reported the greatest increases in comfort in the event of legalization, suggesting that much of Black patients’ current discomfort may be legally motivated. Similarly, Black patients who had used cannabis reported the greatest increases in perceived provider comfort in the event of legalization, though the same did not hold true for Black patients who had *not* used cannabis, who were far more likely to strongly disagree that providers’ comfort discussing cannabis use would increase if cannabis were legal in SC. Taken together, these findings may suggest that while Black patients’ discomfort discussing cannabis with providers may come in large part from concerns about legal repercussions, patients may see providers’ discomfort as stemming from other areas (e.g., stigma, concerns about medication interactions), though this hypothesis and potential explanation requires empirical testing of oncology providers.

Results should be considered within the context of several limitations. First, racial groups were limited to White and Black/African-American participants for analyses, precluding exploration of differences in other racial or ethnic groups. Demographic categorizations were extracted from the EMR and it is possible that patients were mis-categorized or the presented options did not adequately capture the appropriate racial groups. Demographic categories in the EMR were not comprehensive enough to capture within-group variability or other cultural or ethnic factors, which might contribute both to cannabis use and patient-provider relationships. While we acknowledge the importance of studying patient-provider communication as it pertains to cannabis use in other racial groups, we did not have the sample at our cancer center to explore these associations. Second, this study did not collect data from oncology providers or healthcare team members directly, leaving unclear providers’ attitudes, beliefs, and practices regarding their patients’ cannabis use. Patient-provider communication is by definition a two-way street: providers may bring into the conversation their own prior knowledge and biases, which may be of particular relevance in states where cannabis use is not legal and providers may have concerns about patient confidentiality and applicability of medical amnesty laws (which in SC apply only to overdoses and alcohol poisoning; [9]). Due to the lack of provider data, this study was likewise unable to assess the role of provider qualities (such as racial concordance with the patient) in patient-provider communication. Future research is necessary to elucidate the role of providers in promoting honest, open communication about cannabis use with cancer patients. Third, this study was limited to data from SC, where there is no legal cannabis market; results may not generalize to other states with different regulatory frameworks (although recent evidence suggests that even in areas with legal recreational or medical cannabis, illicit markets persist due to barriers of convenience; [10]). Finally, results may in part reflect selection bias, as individuals more comfortable answering questions about cannabis may have been more likely to agree to participate in this survey.

In summary, results of this cross-sectional survey of patients with cancer or survivors in SC highlight comfort, interest, and willingness to discuss cannabis with cancer care providers, particularly among Black patients who already use cannabis. While comfort having cannabis-related discussions was lower among patients who did not endorse cannabis use since their cancer diagnosis, comfort regarding cannabis discussions was neutral on average, suggesting openness to discussions initiated by or led by providers. At the same time, evidence from this study suggests that patients perceive some hesitance on the part of providers that might persist even if cannabis were legalized in SC. These findings warrant further research into providers’ attitudes and perceptions toward cannabis use among cancer patients. This also highlights the need for providers to have evidence-based recommendations and guidelines for patients when cannabis use discussions are initiated.

## Data Availability

No datasets were generated or analysed during the current study.
